# Prevalence and outcomes of proton pump inhibitor associated hypomagnesemia in chronic kidney disease

**DOI:** 10.1371/journal.pone.0197400

**Published:** 2018-05-25

**Authors:** John Hughes, Diana Y. Y. Chiu, Phillip A. Kalra, Darren Green

**Affiliations:** 1 Renal Vascular Research Group, Salford Royal NHS Foundation Trust, Salford, United Kingdom; 2 Institute of Cardiovascular Sciences, University of Manchester, Manchester, United Kingdom; Universidade de Sao Paulo Faculdade de Medicina, BRAZIL

## Abstract

**Background:**

Proton pump inhibitors (PPIs) are one of the most widely prescribed medications across the world. PPIs have been associated with significant electrolyte abnormalities including hypomagnesaemia. We explored the prevalence of PPI associated hypomagnesaemia (PPIH) in different Chronic Kidney Disease (CKD) stages, in different PPI agents, and the impact of PPIH on survival in CKD.

**Methods:**

This was a subgroup analysis of the Salford Kidney Study, a prospective, observational, longitudinal study of non-dialysis CKD patients. Patients with outpatient magnesium samples obtained between 2002 and 2013 were included in the analysis. The prevalence hypomagnesaemia based on mean values over 12 months as well as ‘ever’ hypomagnesaemia were investigated.

**Results:**

1,230 patients were included in this analysis, mean age 64.3± 32.3 years and mean eGFR 29.2±15.8 ml/min/1.73m^2^. Mean serum magnesium in those on PPI was significantly lower than those not on PPI overall (0.85±0.10 mmolL^-1^ versus 0.79±0.12 mmolL^-1^ respectively, p<0.001). This finding was maintained at all CKD stages. The adjusted odds ratio (OR) for mean hypomagnesaemia in PPI use was 1.12 (95% CI 1.06–1.18) p = <0. ‘Ever hypomagnesaemia’ had an OR of 1.12 (95% CI 1.07–1.16) p = <0.001. The expected rise in serum magnesium with declining eGFR was not observed in those on a PPI but was seen in those not on PPI. There was no difference in serum magnesium between PPI drugs. Thiazide diuretics were also associated with hypomagnesaemia independent of PPI use. Cox regression analysis demonstrated no reduction in survival in patients with PPI associated hypomagnesaemia.

**Conclusion:**

No specific PPI drugs show a favourable profile in regards of risk for hypomagnesaemia in CKD. Avoiding concurrent use of PPI and thiazide may be of value in patients with hypomagnesaemia.

## Introduction

Since the introduction of Omeprazole in 1988, Proton-Pump Inhibitors (PPIs) have become the mainstay of treatment for gastroesophageal reflux disease, showing superior efficacy at controlling symptoms, and healing oesophagitis noted on endoscopy when compared to placaebo and H_2_ antagonists (H2A) such as Ranitidine [1],[2],[3]. PPIs are also used for the treatment of gastric and duodenal ulcers, the prevention of non-steroidal anti-inflammatory (NSAID) associated ulcers, and reduction of excessive acid secretion in Zollinger-Ellison syndrome. They are second only to statins in total drug expenditure worldwide at $11 billion [4], with 9.2% of ambulatory United States (US) patients using PPIs [5]. This has led to concerns that there is over use of PPI therapy, with estimates of between 25% and 70% of patients remaining on long term PPI therapy unnecessarily [4],[5],[6]. Not only is this potentially unnecessary prescription of PPIs expensive [6], but it also inappropriately exposes a significant number of people to the side effects of PPI therapy.

The side effects of PPIs are, however, low in prevalence, which itself may be a contributing factor to their over prescription. Reported side effects of PPI therapy include enteric infections such as salmonella and campylobacter [7], clostridium difficile associated diarrhoea [8], community acquired pneumonia [9], hip fractures [10], B12 deficiency [11], neuroendocrine tumours of the stomach [12], drug interactions [13], interstitial nephritis [14] and electrolyte disturbances as such as Hypomagnesaemia and hyponatraemia [4],[12],[15],[16],[17].

Hypomagnesaemia has been shown to be related to the duration the patient is on a PPI, and persists when different PPIs are used [18]. Withdrawal of PPI leads to resolution of hypomagnesaemia but this quickly returns on reintroduction [18]. In haemodialysis (HD) cohorts, PPI users have been repeatedly shown to have a lower serum magnesium than those not on PPIs [19],[20],[21]. This phenomenon occurs despite hypermagnesaemia being commonly observed in HD patients due to the loss of the kidneys ability to excrete magnesium [18],[22]. Importantly, hypomagnesaemia is of clinical significance, being implicated in arterial calcification in renal patients [23], and associated with increased mortality in acutely unwell medical patients [24]. Aberrant magnesium has been associated with other electrolyte abnormalities, cardiac arrhythmias, and a number of neurological and neuromuscular abnormalities [25].

There is a potentially exaggerated importance of adverse outcome associated with PPIH specifically in chronic kidney disease (CKD) patients. The arrhythmic and calcific consequences of hypomagnesaemia are of greater clinical significance in this population because non-atherosclerotic cardiovascular disease, particularly associated with arrhythmia and vascular calcification, is the most common cause of death in CKD. To date the specific impact of proton pump inhibitor associated hypomagnesaemia (PPIH) on mortality in CKD is not known.

The aims of this study were to determine the prevalence of and predictive factors for PPIH in a CKD cohort, to compare prevalence between CKD stages, to establish whether the prevalence varies between specific PPIs, and to determine whether PPIH is associated with all-cause mortality in CKD.

## Method

This was a sub-group analysis of the Salford Kidney Study [26], a single centre prospectively collected observational study of more than 3,000 CKD patients aged ≥18 years and with eGFR <60 mL/min/1.73m^2^, calculated using the 4 variable MDRD formula. The study complies with the declaration of Helsinki and local ethical approval has been obtained (South Manchester Research Ethics Committee, UK, current REC reference 15/NW/0818).

Patients undergo a study-specific additional assessment once a year whilst attending the Nephrology department for an outpatient visit. The information recorded at such visits relevant to this analysis includes extended biochemical profile, co-morbidity and medical event recording, and concurrent medication which includes the use of specific PPI, as well as ranitidine, loop diuretics, aldosterone antagonists, and thiazide diuretics, each of which may also affect serum magnesium.

Patients were included in this analysis who survived at least 12 months from enrolment, and who had at least 12 months follow up thereafter. Patients were enrolled between 2002 and 2013. Patients were selected for this analysis who fell into either of the following categories: a) on PPI at the study visit prior to the first magnesium sample and b) not on PPI at the study visit prior to the first magnesium sample. Patients were excluded if no serum magnesium sample collection was undertaken, if they were undergoing renal replacement therapy (RRT) at the time of magnesium sampling, or if concurrent drug and comorbidity data were not available.

The first outpatient magnesium result was recorded for each patient, alongside further magnesium samples during the following 12 months. Two cohorts were analysed, cohort 1 included the lowest magnesium result for each patient, calculated for every patient in the total population. In this cohort, “ever hypomagnesaemia” was defined as a patient who had any serum magnesium < 0.70 mmolL^-1^ during the 12 months after first sampling. A second cohort, cohort 2 included only those patients with multiple magnesium results available within 12 months of the first magnesium sample, allowing for the calculation of a mean magnesium value over the year. “Mean hypomagnesaemia” was defined as a mean serum magnesium < 0.70 mmolL^-1^ over the 12 months.

All statistical tests were carried out using the R Studio software. The odds ratio for hypomagnesaemia in those patients on PPI versus those not was compared using the *epiR* package to perform a chi square test, and a *lm* package to perform a logistic regression model adjusted for categorical variables CKD stage, co-morbidities, potentially confounding concurrent medication and age group (<60 and >60 years). The mean serum magnesium in patients on and not on PPI were compared using an unpaired t-test. These analyses were performed for all patients and then individually for each of stages 3, 4, and 5 CKD. Serum magnesium was also compared between patients on different PPIs using ANOVA and Tukey HSD to establish any preferential PPI for use in patients at risk of hypomagnesaemia. *Surv*, *ggplot2* and *coxph R* packages were used to perform survival analysis. Follow up was from the date of first serum magnesium level recorded after enrolment until either death or 1^st^ October 2015. Outcome was all-cause mortality. Cox proportional hazard ratios for all-cause mortality in patients with PPI-induced hypomagnesaemia (PPIH) compared to those without PPIH. PPIH was defined as “ever” and “mean hypomagnesaemia” in a patient taking a PPI. Survival analyses were performed for each, adjusted for CKD stage, age group, and concurrent medications and co-morbidities such as diabetes, smoking, and alcohol consumption. Co-variates included in the multivariate model were those that were themselves significant on univariate analysis, or which were deemed to be of potentially significant confounding effect on either serum magnesium or cardiovascular outcome, even if not significant on univariate analysis. Respective examples were the use of thiazide diuretics, and smoking.

## Results

### Population demographics

In Cohort 1, 1,230 patients were included, with a mean age of 64.3 years (standard deviation, SD 32.3) and median eGFR 29.2 ml/min/1.73m^2^ (SD 15.8) (Table 1). There were 469 patients (38.1%) taking PPIs at the time of their first magnesium sample. There was a higher eGFR in those taking a PPI, with a mean eGFR of 30.6 ml/min/1.73m^2^ (S.D 15.8) versus 28.3 ml/min/1.73m^2^ (SD 15.8) in those not on a PPI (p = 0.015, n = 1,230 Table 1). There was a higher prevalence of loop diuretic use in the PPI group with an Odds Ratio (OR) of 1.37 (95% CI 1.09–1.74), p = 0.008 (n = 1,230). This was associated with a greater use of clopidogrel in those on a PPI (OR 2.17 [1.35–3.48] p = 0.001), and a greater use of bisoprolol in those on a PPI (OR 1.53 [1.09–2.14] p = 0.01). No significant association was found between being on a PPI and concurrent aspirin therapy (OR 1.04 [0.82–1.32], p = 0.77). There was a lower prevalence of ranitidine in those on PPIs (OR 0.18 [0.06–0.51], p< 0.001, n = 1,230) (Table 1). 4 patients in the study population were taking magnesium supplements, 2 of whom had an average serum magnesium <0.7 mmolL^-1^, and who were both taking PPI therapy.

**Table 1:** Population demographics.

	Total population	On PPI	Not on PPI (%)	p
N	1,230	469	761	**-**
Age (years)	64.3 ± 32.3	65.0 ± 46.5	64.3 ± 15.7	0.725
Male (%)	62.1	60.3	63.3	0.297
eGFR (mL/min/1.73m2)	29.2 ±15.8	30.6 ±15.8	28.3 ±15.8	0.015
Diabetes (%)	32.7	34.8	31.3	0.219
*Type 1 Diabetes*	*3*.*3*	*4*.*3*	*2*.*6*	0.176
*Type 2 Diabetes*	*9*.*4*	*30*.*5*	*28*.*0*	
Alcohol (units/week)	6.2 ± 10.3	6.0 ± 10.8	6.3 ± 10.0	0.574
Active / ex-smoker (%)	65.0	66.3	63.8	0.290
Loop Diuretics (%)	39.5	44.2	36.6	0.005
Thiazide Diuretics (%)	9.8	10.2	9.6	0.798
MRA (%)	4.2	5.5	3.5	0.120
Ranitidine (%)	3.1	1.2	4.3	0.002
Ethnicity (%)				
*Caucasian*	95.9	96.9	95.3	0.086
*Asian*	3.1	2.0	3.8	
*Black*	0.8	0.8	70.9	
*Other*	0.2	0.4	0.0	
Lives alone (%)	18.6	17.8	19.2	0.615

Key: PPI = proton pump inhibitor, MRA = mineralocorticoid receptor antagonist.

In cohort 2 (n = 609), 244 patients (40.1%) were taking PPIs at the time of their first magnesium sample. There was a higher eGFR in those taking a PPI, with a mean eGFR of 26.9 ml/min/1.73m^2^ (SD 13.7) versus 24.4 ml/min/1.73m^2^ (SD 14.4) in those not on a PPI, (p = 0.034, n = 609). The mean age of those on PPI was 66.1 years (SD 13.2) compared to 63.7 years in those not on a PPI (SD 15.8, p = 0.042). As per cohort 1, there was a lower prevalence of ranitidine in those on PPIs. No different in loop diuretic use was observed.

### Prevalence of mean serum hypomagnesaemia associated with PPI use

In cohort 2 the mean serum magnesium over that year in patients not taking a PPI was 0.85 mmolL^-1^ (SD 0.10) compared to 0.79 mmolL^-1^ (SD 0.12) in those taking a PPI, p<0.001. 45 of 244 patients (18.4%) on PPIs had a mean serum magnesium over 12 months of <0.70 mmolL^-1^ compared to 25 of 365 (6.8%) in those not taking a PPI. This gave an unadjusted OR for mean magnesium of <0.70 mmolL^-1^ of 3.08 (1.83–5.17) in patients on a PPI compared to those not on a PPI (p<0.001). The increased OR persisted in a multivariable logistic regression model correcting for other categorical variables identified on univariate analysis to be predictive of hypomagnesemia (aldosterone antagonist use and CKD stage) with an OR of 1.12 (95% CI 1.06–1.18) p = <0.001 (Table 2). Within the multivariate model, there was also a statistically significant reduction in incidence of hypomagnesaemia in CKD stages 3, 4 and 5 compared to the small cohort of those with CKD 2 (Table 2). No other parameters were significant in univariate analysis. Although inclusion criteria for SKS included eGFR<60mL/min/1.73m^2^, a number of patients demonstrated improvement in eGFR between enrolment and first study visits. It is for this reason that a population of patients with eGFR >60mL/min/1.73m^2^ were able to be included as a reference group for this analysis.

**Table 2:** Odds ratios for mean and ever hypomagnesaemia, demonstrating univariate results for PPI versus not on PPI, and the results of a multivariate model.

	*Mean hypomagnesaemia*			*Ever hypomagnesaemia*		
	OR	95% CI	p value	OR	95% CI	p value
**Univariate**						
On PPI, unadjusted	3.07	1.84–5.23	**<0.001**	2.17	1.61–2.96	**<0.001**
**Multivariate**						
On PPI, adjusted	3.30	1.91–5.86	**<0.001**	2.23	1.62–3.08	**<0.001**
Age >60	1.40	0.76–2.72	0.987	1.03	0.72–1.50	0.85
Ranitidine	2.90	0.62–1.01	0.12	1.04	0.34–2.59	0.93
Loop diuretic	1.13	0.64–1.96	0.67	0.75	0.52–1.06	0.10
MRA	1.65	0.58–4.18	0.31	1.89	0.94–3.61	0.06
On Thiazide	0.88	0.30–2.22	0.81	1.69	1.05–2.66	**0.027**
CKD 3	0.23	0.07–0.74	**0.01**	0.96	0.48–2.08	0.92
CKD 4	0.15	0.05–0.50	**0.001**	0.70	0.35–1.52	0.34
CKD 5	0.19	0.06–0.66	**0.007**	1.23	0.58–2.77	0.60
Non-smoker	0.70	0.38–1.26	0.25	0.81	0.57–1.15	0.24
Female	0.80	0.44–1.40	0.44	1.06	0.76–1.49	0.72
Type 1 Diabetes	0.00	NA–1.65	0.99	1.02	0.36–2.42	0.97
Type 2 Diabetes	1.46	0.83–2.55	0.19	1.67	1.18–2.35	**0.004**
Asian	1.51	0.32–5.22	0.55	1.41	0.54–3.19	0.44
Black	0.00	NA–NA	0.99	1.28	0.19–5.24	0.76
Other	0.00	NA–NA	1.00	5.31	019–141	0.25

Key: PPI = proton pump inhibitor, CKD = chronic kidney disease, OR = odds ratio, MRA = mineralocorticoid receptor antagonist.

### Prevalence of ‘ever hypomagnesaemia’ associated with PPI use

In cohort 1 (n = 1,230) the prevalence of ever having a serum magnesium of < 0.70 mmolL^-1^ amongst those taking a PPI was 108 of 469 (23%) and the prevalence of ever hypomagnesaemia in patients not taking a PPI was 92 of 761 (13%). This gave an unadjusted OR of 2.18 (1.60–2.95) of ever hypomagnesaemia in those on PPI compared to those not on PPI (p < 0.001). In a univariate regression analysis, the OR of ever hypomagnesemia was 1.12 (1.07–1.16) p = <0.001. A multivariate model was performed with other categorical variables which had demonstrated in univariate analysis to be predictive of ever hypomagnesaemia (aldosterone antagonists and thiazide diuretic use, and diabetes, Table 2). The relationship between thiazide diuretic use, type 2 diabetes mellitus as well as PPI therapy with hypomagnesaemia persisted in the multivariate analysis. An association of hypomagnesaemia and aldosterone antagonists was observed in univariate analysis but did not remain significant in a multivariate analysis (Table 2).

### PPI associated hypomagnesaemia across different stages of CKD

**Fig 1:**
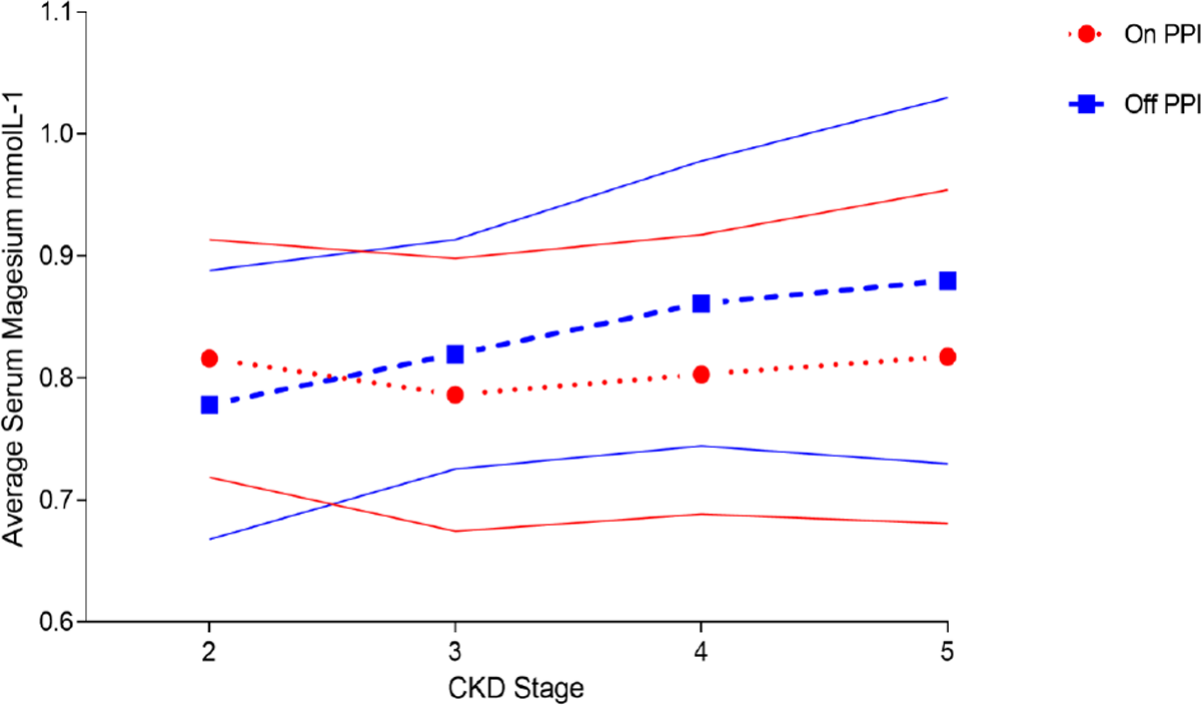
Average serum magnesium increases in patients as CKD progresses in patients NOT on a proton pump inhibitor. This rise in not observed in patients on PPI (error bars demonstrate the standard deviation).

In cohort 2, mean serum magnesium was compared between patients on PPI versus those not on PPI for each stage of CKD. In each of stages 3, 4, and 5, a lower serum magnesium was observed in patients on a PPI (Fig 1). In stage 3 CKD, the mean serum magnesium was 0.78 mmolL^-1^ (SD 0.12) in those on a PPI versus 0.82 mmolL^-1^ (SD 0.08) in those not on a PPI (p = 0.264). In patients with CKD 4 the mean serum magnesium in those taking a PPI was 0.79 mmolL^-1^ (SD 0.11) compared with 0.86 mmolL^-1^ (SD 0.10) in those not on a PPI (p<0.001). In CKD 5 the mean serum magnesium in those taking a PPI was 0.81 mmolL^-1^ (SD 0.14) compared with 0.87 0.81 mmolL^-1^ (SD 0.11) in those not taking a PPI (p = 0.019). In the small number of patients with CK2 (n = 20), there was no significant difference in the mean serum magnesium between those on a PPI and those not on a PPI (0.78 mmolL^-1^ [SD 0.12] and 0.76 mmolL^-1^ [SD 0.10] respectively, p = 0.68). ANOVA demonstrated no significant difference between the mean serum magnesium in patients on PPI across CKD stages (p = 0.67), however a significant difference in the mean serum magnesium in those not taking PPI was observed p = <0.001. The OR of ever hypomagnesaemia in patients using PPI was 2.13 in CKD 3 (n = 166, [1.06–4.28], p = 0.032). In CKD stage 4 (n = 275), the OR was 3.75 [1.92–7.30] p = <0.001), and in CKD 5 (n = 148), the OR was 2.62 [1.15–5.97], p = 0.02).

### Hypomagnesaemia across different PPIs

**Fig 2:**
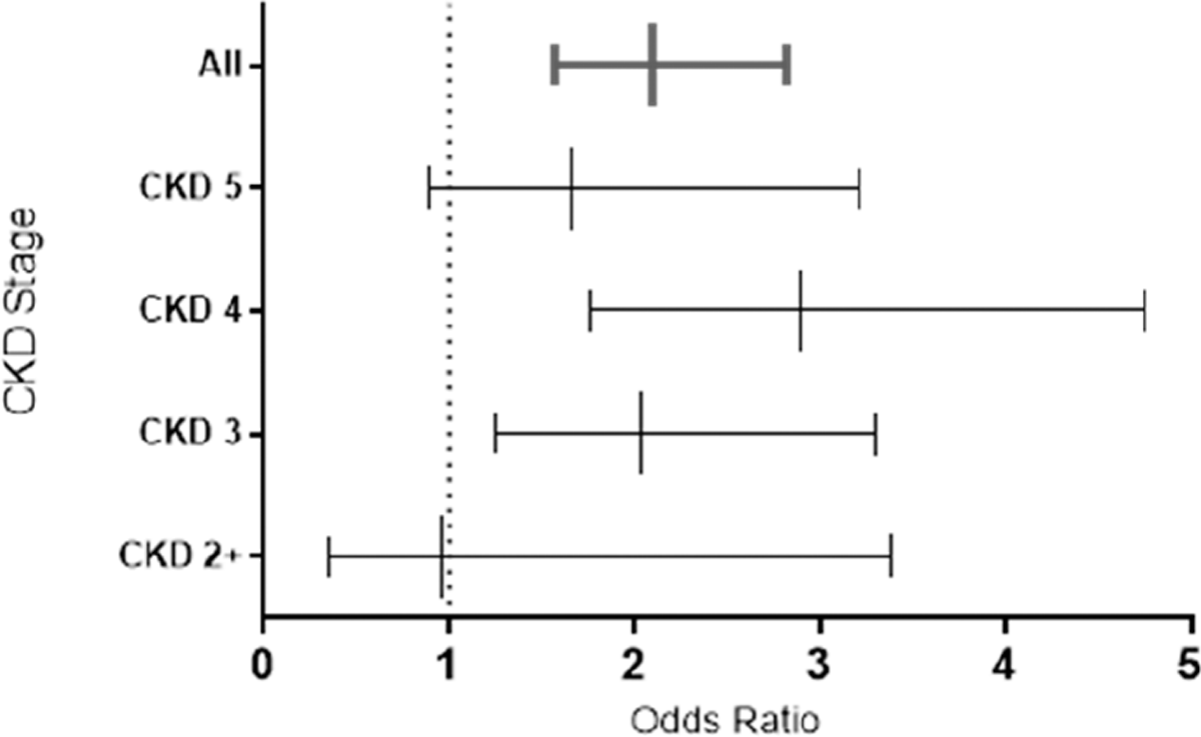
Odds ratios for ever hypomagnesemia in patients on PPI compared to those who are not, separated according to CKD stage.

Of the 244 patients on PPI in cohort 2, 174 were taking Omeprazole (71%), 59 taking Lansoprazole (24%), 5 taking Esomeprazole (2%), 2 taking Pantoprazole (1%) and 4 taking Rabeprazole (2%). The mean serum magnesium in those not taking a PPI and those on each PPI is shown in Fig 2. Mean serum magnesium was 0.85 mmolL^-1^ (SD 0.10) in those not taking a PPI. ANOVA and TukeyHSD have demonstrated that the only statistically significant differences in serum magnesium are between those not on PPI and those on lansoprazole, and those not on PPI and those on Omeprazole. In those taking Omeprazole and not taking a PPI mean serum magnesium of 0.85 mmolL-1 and 0.79 mmolL-1 respectively (p = <0.001) and between those not taking a PPI and Lansoprazole (0.85 mmolL-1 and 0.81 mmolL-1, p = 0.01).

In cohort 2, the OR of mean hypomagnesaemia < 0.70 mmolL^-1^ in patients on Omeprazole compared to no PPI was 3.15 (1.80–5.52), p = <0.001 (PPIH n = 32). For those on lansoprazole the OR of mean hypomagnesaemia < 0.70 mmolL^-1^ was 3.47 (1.64–7.37), p<0.001 (PPIH n = 12). For those on rabeprazole the OR was 4.53 (0.45, 45.19) p = 0.158. Patients taking esomperazole and pantoprazole were excluded because of low numbers (n = 5 and 2 respectively) and no patients with hypomagnesaemia.

In cohort 1, 15 of 469 patients taking PPI (3%) were taking esomeprazole, 112 (24%) taking lansoprazole, 316 (67%) taking omeprazole, 7 (1%) taking pantoprazole and 19 (4%) taking rabeprazole. The unadjusted OR of ever hypomagnesemia in patients taking Omeprazole compared to patients not on a PPI was 2.30 (1.64–3.23) p = <0.001 with 76 patients with hypomagnesaemia on omeprazole. In those taking lansoprazole compared to those not on a PPI the OR was 1.98 (1.20–3.27) p = 0.007, with 24 patients with ever hypomagnesemia. There was no increased OR of ever hypomagnesaemia in those taking esomeprazole, pantoprazole or rabeprazole.

### PPI associated hypomagnesaemia and survival

In cohort 2, survival in the presence of mean PPIH (based on mean serum magnesium less than 0.7 mmolL^-1^ and concurrent PPI) use was compared to patients without hypomagnesaemia irrespective of PPI use. Here, PPIH was not associated with reduced survival based on univariate analysis (HR 1.40 [0.79–2.50], p = 0.25). In a multivariate model including parameters significantly associated with survival on univariate analysis (loop diuretic use, a smoking history, aldosterone antagonists, diabetes, age), PPIH continued to not show an association with survival (HR = 1.08 [0.60–1.94], p = 0.79).

**Fig 3:**
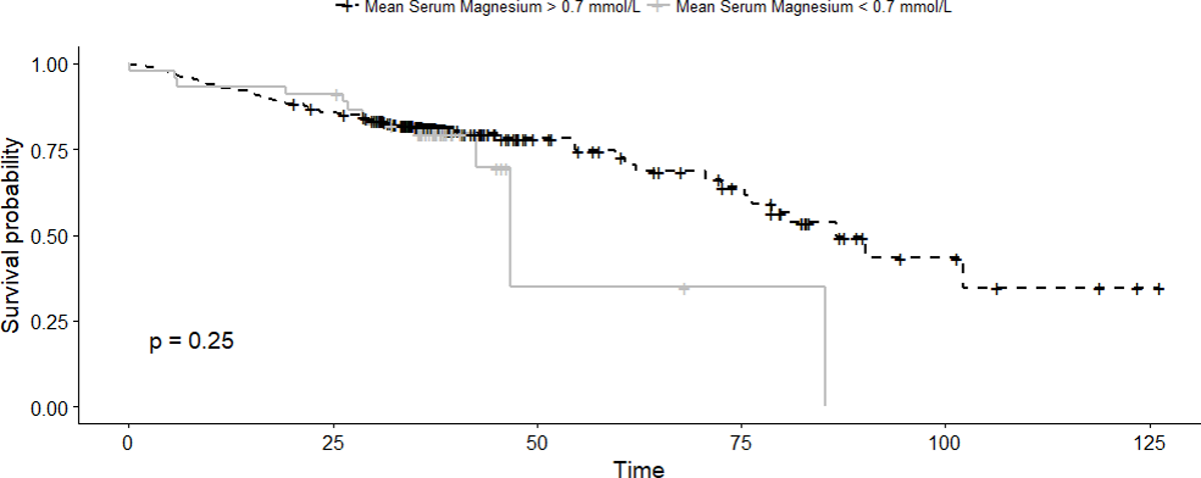
Survival curve for “mean PPIH” versus patients without hypomagnesaemia.

**Fig 4:**
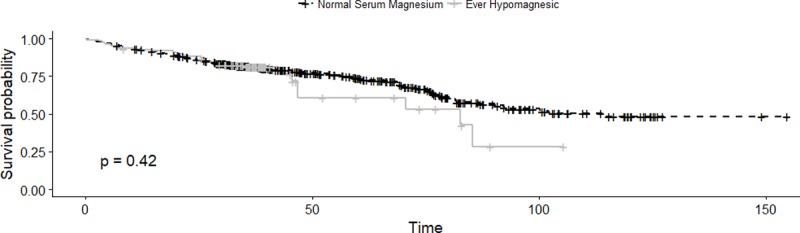
Survival curve for “ever PPIH” versus patients without hypomagnesaemia.

A repeat survival analysis comparing “ever PPIH” versus patients who had never had hypomagnesaemia was performed using patient in cohort 1. The univariate analysis of hazard ratio for death in PPIH also not statistically significant (HR = 1.18 [0.79–1.76], p = 0.42), and again continued to lack significance in a multivariate model. Survival curves for these analyses are shown in Figs 3 and 4.

## Discussion

This observational study demonstrates the high prevalence of PPI usage in CKD, with 39% of patients on PPIs. Patients on a PPI had a slightly higher eGFR, along with an expected discrepancy in ranitidine use, as few patients are on both PPI and H2A therapy. There was also a discrepancy in the use of loop diuretics, with a higher prevalence in those on PPIs, possibly reflecting a higher cardiovascular disease burden in this population. This hypothesis is supported by increased use of clopidogrel and bisoprolol in the PPI cohort.

As expected, and previously shown, there was a significant difference in the mean serum magnesium in patients on and not on a PPI [27], with hypomagnesaemia significantly more common in the patients on PPI. Previous literature demonstrates that PPI associated hypomagnesaemia is related to duration of PPI use and remains low for the duration of PPI therapy. A novel finding of this study is the identification of a further cohort of patients with CKD who are susceptible to sporadic, but not persistent drops in their serum magnesium, of a higher prevalence than an average low magnesium, with 23% having at least one episode of hypomagnesaemia whilst on PPI, and 18% with an average magnesium less than 0.7mmolL^-1^. As hypomagnesaemia is associated with cardiac arrhythmias, the potential importance of one off hypomagnesaemia is highlighted.

A caveat to the above discussion is that mean serum magnesium in patients with CKD stage 2 was actually numerically (although not statistically) higher in those not on a PPI. We can find no strong physiological reason for this given that PPIH is an established phenomenon in the non-CKD population. Many antacid therapies contain magnesium and it may be that these are more commonly used by patients not on PPI, but this argument is speculative at best. We presume this finding in CKD stage 2 to be an artefact of the wide standard deviation seen.

In this population, ever hypomagnesaemia in patients on PPI (i.e. ever PPIH) shows a hazard ratio for mortality of 1.18 (0.82–1.70, p = 0.36). The reason this does not reach statistical significance unlike in other reports on hypomagnesaemia may be because the non-PPIH comparator group in our analyses include patients with hypomagnesaemia, but that which is not associated with PPI use. Persistent PPIH was also not associated with adverse outcome and we presume the reason for this to be similar.

Of note, we found that serum magnesium increased with declining eGFR for patients not on a PPI, but that this effect did not occur in patients on PPI (Fig 1). This is consistent with mechanistic studies. Magnesium homeostasis is maintained through the gastrointestinal (GI) tract and the distal convoluted tubule in the kidney^28^. Active GI absorption of Mg^2+^ occurs via the transient receptor potential melastatin-6 and 7 (TRPM6/7) channels on the apical membrane of the gut enterocyte [28],[29]. Passive Mg^2+^ absorption occurs down the luminal concentration gradient [28]. In the kidney, Mg^2+^ is completely filtered at the glomerulus, with passive reabsorption in the proximal convoluted tubule and the thick ascending limb of the loop of Henle, with active absorption distal convoluted tubule (DCT) via TRPM6 channels [28],[29]. In magnesium deficiency the expression of TRPM6 increases in the DCT, and the expression TRPM6/7 increases in the gut, leading to reduced urinary excretion and increased GI absorption. In PPI induced hypomagnesemia there is an appropriate reduction in urinary elimination of Mg^2+^, implicating the GI tract as the primary culprit [30]. The reduction in the GI luminal pH caused by PPI therapy is thought to reduce the affinity of the TRMP6/7 channels in the gut enterocytes apical wall, reducing active absorption of Mg^2+^ [31]. As CKD progresses, serum Mg^2+^ increases due to the loss of functioning nephrons and reduced urinary elimination, as observed in our population. However, as observed in our population, the decreased gut absorption of Mg^2+^ secondary to PPI usage counteracts the rise in magnesium seen in CKD leading to a lower mean serum magnesium in those on PPI at each stage of CKD.

Our study demonstrates that thiazide diuretics and diabetes are also associated with hypomagnesaemia in CKD. The chronic use of thiazide diuretics may cause a negative balance of potassium, thereby inhibiting distal tubular magnesium uptake and thereby increasing magnesium excretion with resultant measured serum hypomagnesaemia [32]. It may therefore be advisable to limit concurrent PPI and thiazide use in CKD, or to closely monitor serum magnesium in such cases. Extra caution in the use of long term PPI in diabetics may be of value.

We were unable to demonstrate a significant finding favouring a particular PPI with lower risk of PPIH than other PPIs. However, very few patients were on PPI agents other than lansoprazole and omeprazole. It is therefore not possible to extrapolate findings beyond these two drugs. Caution would therefore favour limiting this finding to say that there is no difference in the likelihood of PPIH between either of the two most commonly prescribed drugs, lansoprazole and omeprazole. The small sample sizes for other PPIs is acknowledged as a limitation of this study.

Beyond this, the key limitation of this study is the number of repeat samples available. In the 662 patients the mean number of samples within the first year was 3.3. This likely reflects the serum magnesium as a test was removed from the parameters included in the standard “renal biochemistry” profile undertaken by the Study reference laboratory during the Salford Kidney Study. The findings of this study and others do favour magnesium being monitored more closely than perhaps is the norm in nephrology outpatient settings. With regard to the findings of this analysis, it may also be that some results included in this study were requested for clinical reasons outside of the study. Therefore, there may be a higher prevalence of Hypomagnesaemia in the samples than would be seen in the stable CKD cohort.

## Conclusion

This study highlights the importance of rational prescribing of PPIs in CKD, given the high risk of hypomagnesaemia seen. These low toxicity medications can have subtle undetected effects which may be associated with adverse outcome in a complex patient group.

In summary, the key points of this study are:

- Hypomagnesaemia is no more or less likely to occur with the use of specific PPIs.
- Serum magnesium does not increase with declining eGFR if a patient is taking a PPI, but does if a patient is not taking a PPI.
- Thiazide diuretics were also associated with hypomagnesaemia and so avoiding concurrent use of these drugs with PPI may aid in reducing the burden of hypomagnesaemia.
- Patients with diabetes were more likely to have hypomagnesaemia. More careful monitoring of serum magnesium may be advisable in this patient population.
